# Supplementary material for: Pitfalls in using phenanthroline to study the causal relationship between promoter nucleosome acetylation and transcription
Source: Nat Commun. 2022 Jun 29;13:3726. doi: 10.1038/s41467-022-30350-3 (PMC9242984; doi:10.1038/s41467-022-30350-3)
Supplement: Supplementary file 1 — Description of additional Supplementary File [file 41467_2022_30350_MOESM1_ESM.pdf]

Descriptions of Additional Supplementary Files

_Supplementary Data1: Gene name (name), Gene Ontology terms and pvalues of gene groups that are the most downregulated (fold change >- 2.75 (Log2); n=268, blue) or the least affected (fold change <- 0.32 (log2); n=120) following 1,10-pt treatment. Fold change is determined by comparing Rpb3 ChIP-seq signal at promoters of all genes (n=5045) before and after treatment.

Supplementary Data2: Gene name (name), Gene Ontology terms and pvalues of gene groups where Epl1 ChIP-seq signal quantified in a window of 100 base pairs (bp) centered on the Epl1 peaks increases >1,2 fold or decrease
