# Supplementary material for: Pitfalls in using phenanthroline to study the causal relationship between promoter nucleosome acetylation and transcription
Source: Nat Commun. 2022 Jun 29;13:3726. doi: 10.1038/s41467-022-30350-3 (PMC9242984; doi:10.1038/s41467-022-30350-3)
Supplement: Supplementary file 4 — Reporting Summary [file 41467_2022_30350_MOESM4_ESM.pdf]

Corresponding author(s): ALBERT Benjamin
SHORE David

Last updated by author(s): Mar 7, 2022

Reporting Summary

Nature Portfolio wishes to improve the reproducibility of the work that we publish. This form provides structure for consistency and transparency in reporting. For further information on Nature Portfolio policies, see our Editorial Policies and the Editorial Policy Checklist.

Statistics

For all statistical analyses, confirm that the following items are present in the figure legend, table legend, main text, or Methods section.

n/a	Confirmed	
x		The exact sample size (*n*) for each experimental group/condition, given as a discrete number and unit of measurement
	x	A statement on whether measurements were taken from distinct samples or whether the same sample was measured repeatedly
	x	The statistical test(s) used AND whether they are one- or two-sided<br>*Only common tests should be described solely by name; describe more complex techniques in the Methods section.*
x		A description of all covariates tested
x		A description of any assumptions or corrections, such as tests of normality and adjustment for multiple comparisons
	x	A full description of the statistical parameters including central tendency (e.g. means) or other basic estimates (e.g. regression coefficient) AND variation (e.g. standard deviation) or associated estimates of uncertainty (e.g. confidence intervals)
	x	For null hypothesis testing, the test statistic (e.g. *F*, *t*, *r*) with confidence intervals, effect sizes, degrees of freedom and *P* value noted<br>*Give P values as exact values whenever suitable.*
x		For Bayesian analysis, information on the choice of priors and Markov chain Monte Carlo settings
x		For hierarchical and complex designs, identification of the appropriate level for tests and full reporting of outcomes
	x	Estimates of effect sizes (e.g. Cohen's *d*, Pearson's *r*), indicating how they were calculated

Our web collection on statistics for biologists contains articles on many of the points above.

Software and code

Policy information about availability of computer code

Data collection: Data was collected using standard Illumina software for the NextSeq 500 and HiSeq 2500 platforms.

Data analysis:

The Integrative Genomics Viewer (IGV) was used for the visual exploration of genomic data (James et al. 2011) in Figure 1A,B,C,D and Figure 2E.

ChIP-seq datasets (Replicate 1) from 1,10-pt-treated or untreated cells were normalized to silent regions by ChIP Normalization Factors calculated in Martin et al. 2021 1. Rpb3 ChIP-seq signal was quantified for each promoter defined as the 400 bp region upstream of the transcription start site (TSS), as obtained from 15. To calculate ChIP-seq signals fold change, a ratio between the total number of reads for each promoter from 1,10-pt treated cells and the number of reads from untreated cells was made. RP gene list was defined in Zencir et al. 2020 and Hsf1 gene list in Pincus et al. 2018.

Values for all correlation measurements are the Pearson's correlation coefficients.

Aggregation plot (Figure 2B) tool for signal profiling centered on Epl1 peaks defined by Martin et al. 2021 were generated using the sitepro script (version 1.0.0) from Galaxy) (Liu et al. 2011). Data are taken from replicate 1 from Martin et al. 2021.
Ep1 and Epl1(1-485) ChIP-seq signal was quantified in Figure 2A and 2C for each promoter defined as the 100 bp region upstream and downstream of Epl1 peaks defined by Martin et al. 2021. In the box plots, the box shows the 25th–75th percentile, whiskers show the 10th–90th percentile, and dots show the 5th and 95th percentiles. Statistical significance of difference between groups was evaluated using the

Wilcoxon test.

Yeast Mine (https://yeastmine.yeastgenome.org/yeastmine/begin.do) was used to determine GO-terms and MEME (Bailey et al, 2009) was used for motif enrichment analysis, with the following settings: anr -nmotifs 5 -minw 6 -maxw 20 -objfun classic -revcomp -markov_order 0.

For manuscripts utilizing custom algorithms or software that are central to the research but not yet described in published literature, software must be made available to editors and reviewers. We strongly encourage code deposition in a community repository (e.g. GitHub). See the Nature Portfolio guidelines for submitting code & software for further information.

Data

Policy information about availability of data

All manuscripts must include a data availability statement. This statement should provide the following information, where applicable:

- Accession codes, unique identifiers, or web links for publicly available datasets
- A description of any restrictions on data availability
- For clinical datasets or third party data, please ensure that the statement adheres to our policy

Data used in this study are from the NCBI Gene Expression Omnibus under the following accession codes: GSE110287, RNAPII ChIP-seq±1,10-pt, Epl1 and H3K23ac/H4K8ac ChIP-seq±1,10-pt; GSE110286, Rpb3 ChIP-seq; GSE125226, Serine-5 Rpb1 ChIP-seq ± Heat Shock; GSE20870, Tbf1 ChIP-seq; GSE61596, Rap1 ChIP-seq.

Field-specific reporting

Please select the one below that is the best fit for your research. If you are not sure, read the appropriate sections before making your selection.

- [x] Life sciences
- [ ] Behavioural & social sciences
- [ ] Ecological, evolutionary & environmental sciences

For a reference copy of the document with all sections, see nature.com/documents/nr-reporting-summary-flat.pdf

Life sciences study design

All studies must disclose on these points even when the disclosure is negative.

Sample size	Sample sizes for ChIPseq experiments was limited to two replicates due to financial constraints as explained in the original study (Martin et al. 2021, (PMID: 33431884))
Data exclusions	For analysis of epl1(1-485) data reads of chrXII were removed from all data sets as done by Martin et al. 2021.
Replication	The reproducibility for analyses was confirmed by analysis of two independent experiments except for the MNase Epl1 and Epl1(1-485) ChIP-seq experiments. For these, only one replicate was performed in the original study (Martin et al. 2021, (PMID: 33431884)).
Randomization	As explained by Martin et al. 2021 (PMID: 33431884), all experiments were performed with large numbers of isogenic cells and thus randomization was not required.
Blinding	As explained by Martin et al. 2021 (PMID: 33431884), blinding was not required.

Reporting for specific materials, systems and methods

We require information from authors about some types of materials, experimental systems and methods used in many studies. Here, indicate whether each material, system or method listed is relevant to your study. If you are not sure if a list item applies to your research, read the appropriate section before selecting a response.

Materials & experimental systems

n/a	Involved in the study	
x		Antibodies
x		Eukaryotic cell lines
x		Palaeontology and archaeology
x		Animals and other organisms
x		Human research participants
x		Clinical data
x		Dual use research of concern

Methods

n/a	Involved in the study	
	x	ChIP-seq
x		Flow cytometry
x		MRI-based neuroimaging

ChIP-seq

Data deposition

☒ Confirm that both raw and final processed data have been deposited in a public database such as GEO.

☒ Confirm that you have deposited or provided access to graph files (e.g. BED files) for the called peaks.

Data access links
May remain private before publication.

https://www.ncbi.nlm.nih.gov/geo/query/acc.cgi?acc=GSE110287 All files are deposited by Martin BJ, Howe LJ

Files in database submission

GSM2985387_pt15_rep1_input_coverage_per_mil_frag.wig
GSM2985412_EPL1HA6_rep1_tp0_100uMNase_H4K8ac_coverage_per_mil_frag.wig
GSM2985413_EPL1HA6_rep2_tp0_100uMNase_H4K8ac_coverage_per_mil_frag.wig
GSM2985414_EPL1HA6_rep1_tp_pt15_100uMNase_H4K8ac_coverage_per_mil_frag.wig
GSM2985415_EPL1HA6_rep2_tp_pt15_100uMNase_H4K8ac_coverage_per_mil_frag.wig
GSM2985416_EPL1HA6_rep1_tp0_100uMNase_Input_coverage_per_mil_frag.wig
GSM2985417_EPL1HA6_rep2_tp0_100uMNase_Input_coverage_per_mil_frag.wig
GSM2985418_EPL1HA6_rep1_tp_pt15_100uMNase_Input_coverage_per_mil_frag.wig
GSM2985419_EPL1HA6_rep2_tp_pt15_100uMNase_Input_coverage_per_mil_frag.wig
GSM2985433_EPL1HA6_rep1_tp0_Sonicated_HA_IP_coverage_per_mil_frag.wig
GSM2985434_EPL1HA6_rep2_tp0_Sonicated_HA_IP_coverage_per_mil_frag.wig
GSM2985435_EPL1HA6_rep1_tp_pt15_Sonicated_HA_IP_coverage_per_mil_frag.wig
GSM2985436_EPL1HA6_rep2_tp_pt15_Sonicated_HA_IP_coverage_per_mil_frag.wig
GSM2985437_EPL1HA6_rep1_tp0_Sonicated_Input_coverage_per_mil_frag.wig
GSM2985438_EPL1HA6_rep2_tp0_Sonicated_Input_coverage_per_mil_frag.wig
GSM2985439_EPL1HA6_rep1_tp_pt15_Sonicated_Input_coverage_per_mil_frag.wig
GSM2985440_EPL1HA6_rep2_tp_pt15_Sonicated_Input_coverage_per_mil_frag.wig
GSM4100666_EPL1_rep1_tp0_100uMNase_HA_IP_coverage_per_mil_frag.wig
GSM4100667_epl1.485_rep1_tp0_100uMNase_HA_IP_coverage_per_mil_frag.wig
GSM4100668_epl1.485_rep1_tp0_100uMNase_Input_coverage_per_mil_frag.wig
GSM4100669_epl1.485_6HA_rep1_tp0_Sonicated_HA_IP_coverage_per_mil_frag.wig
GSM4100670_epl1.485_6HA_rep1_tp0_Sonicated_Input_coverage_per_mil_frag.wig
GSM4100671_epl1.485_6HA_rep1_tp_pt15_Sonicated_HA_IP_coverage_per_mil_frag.wig
GSM4100672_epl1.485_6HA_rep1_tp_pt15_Sonicated_Input_coverage_per_mil_frag.wig
GSM4100673_epl1.485_6HA_rep2_tp0_Sonicated_HA_IP_coverage_per_mil_frag.wig
GSM4100674_epl1.485_6HA_rep2_tp0_Sonicated_Input_coverage_per_mil_frag.wig
GSM4100675_epl1.485_6HA_rep2_tp_pt15_Sonicated_HA_IP_coverage_per_mil_frag.wig
GSM4100676_epl1.485_6HA_rep2_tp_pt15_Sonicated_Input_coverage_per_mil_frag.wig
GSM4849495_epl1.485HA6_rep1_tp0_100uMNase_H4K8ac_coverage_per_mil_frag.wig
GSM4849496_epl1.485HA6_rep2_tp0_100uMNase_H4K8ac_coverage_per_mil_frag.wig
GSM4849497_epl1.485HA6_rep1_pt15_100uMNase_H4K8ac_coverage_per_mil_frag.wig
GSM4849498_epl1.485HA6_rep2_pt15_100uMNase_H4K8ac_coverage_per_mil_frag.wig
GSM4849499_epl1.485HA6_rep2_tp0_100uMNase_Input_coverage_per_mil_frag.wig
GSM4849500_epl1.485HA6_rep1_pt15_100uMNase_Input_coverage_per_mil_frag.wig
GSM4849501_epl1.485HA6_rep2_pt15_100uMNase_Input_coverage_per_mil_frag.wig
GSM4849502_Untagged_100uMNase_HA_IP_coverage_per_mil_frag.wig
GSM4849503_Untagged_100uMNase_Input_coverage_per_mil_frag.wig
GSM4850569_tp0_rep1_H3K23ac_coverage_per_mil_frag.wig GSM4850570_tp0_rep1_Input_coverage_per_mil_frag.wig
GSM4850571_tp0_rep2_H3K23ac_coverage_per_mil_frag.wig GSM4850572_tp0_rep2_Input_coverage_per_mil_frag.wig
GSM4850573_tsa15_rep1_H3K23ac_coverage_per_mil_frag.wig
GSM4850574_tsa15_rep1_Input_coverage_per_mil_frag.wig
GSM4850575_tsa15_rep2_H3K23ac_coverage_per_mil_frag.wig
GSM4850576_tsa15_rep2_Input_coverage_per_mil_frag.wig EPL1_rep1_tp0_100uMNase_HA_IP_R1.fastq
EPL1_rep1_tp0_100uMNase_HA_IP_R2.fastq epl1.485_6HA_rep1_tp_pt15_Sonicated_HA_IP_R1.fastq
epl1.485_6HA_rep1_tp_pt15_Sonicated_HA_IP_R2.fastq epl1.485_6HA_rep1_tp_pt15_Sonicated_Input_R1.fastq
epl1.485_6HA_rep1_tp_pt15_Sonicated_Input_R2.fastq epl1.485_6HA_rep1_tp0_Sonicated_HA_IP_R1.fastq
epl1.485_6HA_rep1_tp0_Sonicated_HA_IP_R2.fastq epl1.485_6HA_rep1_tp0_Sonicated_Input_R1.fastq
epl1.485_6HA_rep1_tp0_Sonicated_Input_R2.fastq epl1.485_6HA_rep2_tp_pt15_Sonicated_HA_IP_R1.fastq
epl1.485_6HA_rep2_tp_pt15_Sonicated_HA_IP_R2.fastq epl1.485_6HA_rep2_tp_pt15_Sonicated_Input_R1.fastq
epl1.485_6HA_rep2_tp_pt15_Sonicated_Input_R2.fastq epl1.485_6HA_rep2_tp0_Sonicated_HA_IP_R1.fastq
epl1.485_6HA_rep2_tp0_Sonicated_HA_IP_R2.fastq epl1.485_6HA_rep2_tp0_Sonicated_Input_R1.fastq
epl1.485_6HA_rep2_tp0_Sonicated_Input_R2.fastq epl1.485_rep1_tp0_100uMNase_HA_IP_R1.fastq
epl1.485_rep1_tp0_100uMNase_HA_IP_R2.fastq epl1.485_rep1_tp0_100uMNase_Input_R1.fastq
epl1.485_rep1_tp0_100uMNase_Input_R2.fastq epl1.485HA6_rep1_pt15_100uMNase_H4K8ac_R1.fastq
epl1.485HA6_rep1_pt15_100uMNase_H4K8ac_R2.fastq epl1.485HA6_rep1_pt15_100uMNase_Input_R1.fastq
epl1.485HA6_rep1_pt15_100uMNase_Input_R2.fastq epl1.485HA6_rep1_tp0_100uMNase_H4K8ac_R1.fastq
epl1.485HA6_rep1_tp0_100uMNase_H4K8ac_R2.fastq epl1.485HA6_rep2_pt15_100uMNase_H4K8ac_R1.fastq
epl1.485HA6_rep2_pt15_100uMNase_H4K8ac_R2.fastq epl1.485HA6_rep2_pt15_100uMNase_Input_R1.fastq
epl1.485HA6_rep2_pt15_100uMNase_Input_R2.fastq epl1.485HA6_rep2_tp0_100uMNase_H4K8ac_R1.fastq
epl1.485HA6_rep2_tp0_100uMNase_H4K8ac_R2.fastq epl1.485HA6_rep2_tp0_100uMNase_Input_R1.fastq
epl1.485HA6_rep2_tp0_100uMNase_Input_R2.fastq EPL1HA6_rep1_tp_pt15_100uMNase_H4K8ac_R1.fastq
EPL1HA6_rep1_tp_pt15_100uMNase_H4K8ac_R2.fastq EPL1HA6_rep1_tp_pt15_100uMNase_Input_R1.fastq
EPL1HA6_rep1_tp_pt15_100uMNase_Input_R2.fastq EPL1HA6_rep1_tp_pt15_Sonicated_HA_IP_R1.fastq

EPL1HA6_rep1_tp_pt15_Sonicated_HA_IP_R2.fastq EPL1HA6_rep1_tp_pt15_Sonicated_Input_R1.fastq
EPL1HA6_rep1_tp_pt15_Sonicated_Input_R2.fastq EPL1HA6_rep1_tp0_100uMNase_H4K8ac_R1.fastq
EPL1HA6_rep1_tp0_100uMNase_H4K8ac_R2.fastq EPL1HA6_rep1_tp0_100uMNase_Input_R1.fastq
EPL1HA6_rep1_tp0_100uMNase_Input_R2.fastq EPL1HA6_rep1_tp0_Sonicated_HA_IP_R1.fastq
EPL1HA6_rep1_tp0_Sonicated_HA_IP_R2.fastq EPL1HA6_rep1_tp0_Sonicated_Input_R1.fastq
EPL1HA6_rep1_tp0_Sonicated_Input_R2.fastq
EPL1HA6_rep2_tp_pt15_100uMNase_H4K8ac_R1.fastq EPL1HA6_rep2_tp_pt15_100uMNase_H4K8ac_R2.fastq
EPL1HA6_rep2_tp_pt15_100uMNase_Input_R1.fastq EPL1HA6_rep2_tp_pt15_100uMNase_Input_R2.fastq
EPL1HA6_rep2_tp_pt15_Sonicated_HA_IP_R1.fastq EPL1HA6_rep2_tp_pt15_Sonicated_HA_IP_R2.fastq
EPL1HA6_rep2_tp_pt15_Sonicated_Input_R1.fastq EPL1HA6_rep2_tp_pt15_Sonicated_Input_R2.fastq
EPL1HA6_rep2_tp0_100uMNase_H4K8ac_R1.fastq EPL1HA6_rep2_tp0_100uMNase_H4K8ac_R2.fastq
EPL1HA6_rep2_tp0_100uMNase_Input_R1.fastq EPL1HA6_rep2_tp0_100uMNase_Input_R2.fastq

Genome browser session
(e.g. UCSC)

Please refer to wig files at https://www.ncbi.nlm.nih.gov/geo/query/acc.cgi?acc=GSE110287

Methodology

Replicates

The reproducibility for analyses was confirmed by analysis of two independent experiments except for the MNase Epl1 and Epl1 (1-485) ChIP-seq experiments. For these, only one replicate was performed in the original study (Martin et al. 2021, (PMID: 33431884)).

Sequencing depth

Sample, Mapped Reads, Uniquely Mapped Reads
EPL1-6HA_MNase_HA_IP_tp0, 13662338, 12318028
epl1.485-6HA_MNase_HA_IP_tp0, 18180146, 16784518
EPL1-6HA_Sonic_HA_IP_pt15_rep1, 7766190, 7529484
EPL1-6HA_Sonic_HA_IP_pt15_rep2, 4156226, 4084592
EPL1-6HA_Sonic_HA_IP_tp0_rep1, 4652560, 4567586
EPL1-6HA_Sonic_HA_IP_tp0_rep2, 9314654, 9035532
epl1.485-6HA_Sonic_HA_IP_pt15_rep1, 3710104, 3658112
epl1.485-6HA_Sonic_HA_IP_pt15_rep2, 3249480, 3198912
epl1.485-6HA_Sonic_HA_IP_tp0_rep1, 4777056, 4691198
epl1.485-6HA_Sonic_HA_IP_tp0_rep2, 3165104, 3126848
Untagged_Sonic_HA_IP_tp0, 7342588, 6915818
H3K23ac_pt15_rep1, 5085478, 4928616
H3K23ac_pt15_rep2, 19940100, 19058578
H3K23ac_tp0_rep1, 18627464, 17938562
H3K23ac_tp0_rep2, 16525690, 15952832
H3K23ac_tsa15_rep1, 18677944, 18037672
H3K23ac_tsa15_rep2, 15002682, 14578530
H4K12ac_pt15_rep1, 15903400, 15436774
H4K12ac_pt15_rep2, 18761686, 18219906
H4K12ac_tp0_rep1, 16730100, 16256180
H4K12ac_tp0_rep2, 14442902, 13939340
H4K12ac_tsa15_rep1, 18485180, 17820332
H4K12ac_tsa15_rep2, 15710962, 15219514
EPL1-6HA_MNase_H4K8ac_IP_pt15_rep1, 17754046, 16922200
EPL1-6HA_MNase_H4K8ac_IP_pt15_rep2, 19216428, 18133552
EPL1-6HA_MNase_H4K8ac_IP_tp0_rep1, 9262270, 8953380
EPL1-6HA_MNase_H4K8ac_IP_tp0_rep2, 19195174, 17846466
EPL1-6HA_MNase_Input_pt15_rep1, 6508738, 6327076
EPL1-6HA_MNase_Input_pt15_rep2, 10003528, 9647302
EPL1-6HA_MNase_Input_tp0_rep1, 8025918, 7709658

Antibodies

Refer to Martin et al. 2021, (PMID: 33431884).

Peak calling parameters

Refer to Martin et al. 2021, (PMID: 33431884)

Data quality

Refer to Martin et al. 2021, (PMID: 33431884).

Software

Data was collected using standard Illumina software for the nextSeq500 and HiSeq 2500 platforms.
